# Henry et al. (2012) homing failure formula, assumptions, and basic mathematics: a comment

**DOI:** 10.3389/fphys.2013.00142

**Published:** 2013-06-20

**Authors:** David Guez

**Affiliations:** Faculty of Science and IT, The University of NewcastleCallaghan, NSW, Australia

In March 2012 Henry et al. published a paper that explored whether or not the consumption of thiamethoxam via nectar could be a causal factor of Colony Collapse Disorder (CCD) in honeybees. In the first part of their report, Henry et al. (2012) measured the homing success after “ecologically relevant” thiamethoxam exposure and compared it to non-thiamethoxam exposed, control homing rates [see Guez (2013) for a critique of this aspect of their work]. In the second part of their report, they applied their homing study results to the honeybee population dynamic model devised by Khoury et al. (2011), and from that they concluded that dietary thiamethoxam intoxication may potentially contribute to CCD.

Khoury et al.'s (2011) model is build upon the hypothesis that colony failure occurs when bee death rate become unsustainable at the colony level, and the salient assumption that mortality within the hive is negligible. Khoury et al.'s (2011) model allows the evolution of the honeybee hive population to be projected over time. Model outputs are dependent on the total hive population (at the start, and then at any given time), the queen's egg laying rate (L), an eclosion rate that is directly dependent upon the hive population and modulated via the parameter *w* (the larger *w* the lower the eclosion rate), and the forager mortality rate or forager homing failure (*m*). In Khoury et al.'s (2011) model therefore, the population growth of the colony is controlled mainly by the parameters *L* and *w* [but see Cresswell and Thompson (2012) for a critique of the choice of *w*], whereas population decline is dependent on *m*, the forager mortality rate or forager homing failure.

In order to model the population dynamic under dietary thiamethoxam exposure, Henry et al.'s (2012) undertaking was to calculate the homing failure due to pesticide exposure (*m*_hf_). *m*_hf_ was then used to increase the value of *m* for population projection under a dietary thiamethoxam exposed scenario, compared to the “normal” homing failure *m* postulated in the non-thiamethoxam exposed scenario. In my previous critic of Henry et al. (2012) I pointed out that the way in which Henry et al. calculated *m*_hf_ was incorrect given the authors claim that *m*_hf_ “[…] estimates the proportion of exposed foragers that might disappear due solely to post-exposure homing failure, all other sources of mortality or homing failure set apart (natural mortality, predation, manipulation stress).”

In their answer to my critic (Guez, 2013) Henry and Decourtye (2013) maintain that the formula used in Henry et al. (2012) to calculate homing failure in honeybees post pesticide exposure (*m*_hf_) is correct. In this note I show that the calculated *m*_hf_ value is largely impacted by assumptions, and that regardless of which assumption is taken, Henry et al. (2012) formula for calculating honeybee homing failure post-pesticide exposure is incorrect.

In their original report Henry et al. (2012) propose that:

(1) $$m_{\text{hf}}=(\left[\text{Homing success of the control}\right]-[\text{Homing success of the treatment}])/[\text{Homing success of the control}]$$

whereas Guez (2013) contends that it should be:

(2) $$m_{\text{hf}}=([\text{Homing success of the control}]-[\text{Homing success of the treatment}]).$$

To evaluate the validity of the formula used to calculate *m*_hf_ it is important to understand that *m*_hf_ is used to estimate the *m* parameter of the Khoury et al. (2011) population dynamic model after pesticide intoxication. The parameter *m* represents the attrition of foragers with time and is expressed in individuals.day^−1^. In the absence of pesticide exposure Henry et al. (2012) fixed this parameter to *m* = 0.154 individuals.day^−1^, assuming an expected forager lifespan of about 6.5 days in line with previously published results [see Henry et al. (2012) for details]. However, with pesticide exposure, it is difficult to discern exactly how the *m* parameter was estimated in Henry et al.'s (2012) original publication.

In their supplemental material Henry et al. (2012) write as follows (emphasis added):

We ran simulations under the hypotheses of (i) constant forager death rate with no forager exposure, and (ii) forager death rate raised by post-exposure homing failure *m*_hf_ during a 30-days oilseed rape flowering period […]. In the later configuration, **exposed foragers were assigned a probability of disappearance combining daily death rate and the additional mortality due solely to post-exposure homing failure**.

From this statement it is clear that: (i) *m*_hf_ was used by Henry et al. (2012) to raise the homing failure rate after pesticide exposition, and (ii) *m*_hf_ represents the post-exposure homing failure.

## Using the *m*_hf_ formula put forward by Henry et al. (2012) and Henry and Decourtye (2013)

If we use the equation put forward by Henry et al. (2012) (Equation 1), *m*_hf_ is expressed as a proportional decrease in homing success in the treatment group relative to the control, as highlighted in the comment of Henry and Decourtye (2013). In the case of the upper bound *m*_hf_ determined in Experiment 2, Henry et al. (2012) found that there was 0.316 less homing success in the treatment group than in the control group. In other words, there was 31.6% less homing success with treatment. However, since the parameter *m* of Khoury et al. (2011) is intended to represent the attrition of foragers with time, what is needed is the proportional increase or decrease in homing failure of the treatment given the control, not the decrease in homing success of the treatment given the control. As counterintuitive as it may be, these two values (i.e., the proportional increase in homing failure and the proportional decrease in homing success) are not numerically equivalent.

Taking Experiment 2 in Henry et al. (2012) as an example, homing success was reported as 0.83 and 0.57 individuals.day^−1^ in the respective control and treatment groups, meaning that 0.316 less individuals.day^−1^ returned to their hive in the treatment group relative to the control. Indeed, [treatment homing success] = 0.83 − (0.83 × 0.316) = 0.57 individuals.day^−1^. However, these homing success rates also mean that 0.17 and 0.43 individuals.day^−1^ failed to return in the respective control and treatment groups ([homing failure] = 1 − [homing success]). This translates into a 1.55 fold (155%) additional increase in homing failure rate relative to the control (0.17 individuals.day^−1^), and represents a treatment homing failure of 0.43 individuals.day^−1^:

$$\begin{array}{l} \left[\text{Treatment homing failure}\right]\\ =[\text{Control homing failure}]\\ \;+([\text{Control homing failure}]\times 1.55)\\ =(0.17+(0.17\times 1.55))\\ =0.43\text{ individuals}.{\text{day}}^{-\text{1}}. \end{array}$$

If *m*_hf_ expresses the proportional increase in homing failure in the treatment given the control it should be calculated as follows:

(3^1^) $$m_{\text{hf}}=([\text{Treatment homing failure}]-[\text{Control homing failure}])/[\text{Control homing failure}],$$

and not as proposed by Henry et al. (2012):

(1) $$m_{\text{hf}}=([\text{Homing success of the control}]-[\text{Homing success of the treatment}])/[\text{Homing success of the control}].$$

Therefore, *m*_hf_ should equal 0.59 and 1.55 for Experiments 1 and 2 respectively, not 0.102 and 0.310 as stated in Henry et al. (2012). The *m*_hf_ values reported by Henry et al. (2012) are therefore erroneous and stem from the use of an incorrect formula that calculates the proportional decrease in homing success instead of a proportional increase in homing failure in the treatment relative to the control. In their supplemental material Henry et al. (2012) claim that the *m*_hf_ value they calculated **“*[…] estimates the proportion of exposed foragers that might disappear due solely to post-exposure homing failure, all other sources of mortality or homing failure set apart (natural mortality, predation, manipulation stress)*”** (emphasis added). On the contrary, it assumes that the additional mortality due to pesticide exposure is proportional to all other sources of mortality or homing failure. This is also regardless of the fact that Henry et al. (2012) failed to note that the proportional decrease in homing success was not equivalent to the proportional increase in homing failure in the treatment given the control. In fact, if *m*_hf_ is expressed as in Equation 3, we assume that the additional homing failure with pesticide exposure is proportional to the observed, non-exposed homing failure. The same is true if we erroneously use Equation 1 to calculate *m*_hf_ as Henry et al. (2012). Thus, if *m*_hf_ is expressed as a proportional increase of homing failure give the “normal” attrition rate, it impacts upon how the *m* parameter of Khoury et al. (2011) need to be estimated:

$$\begin{array}{l} m_{\text{pesticide}}=m_{\text{non-exposure}}\\ \;+(m_{\text{non-exposure}}\times m_{\text{hf}})\\ \text{ individuals}.{\text{day}}^{-1}\\ \;(\text{with }m_{\text{hf}}\text{ given by Equation 3}). \end{array}$$

Therefore, the *m* parameter of Khoury et al.'s (2011) population dynamic model should be (*m*_hf_ × 100%) larger following pesticide exposure than the *m* parameter that is used under non pesticide exposure conditions.

## Using the *m*_hf_ formula put forward by Guez (2013)

The homing failure of the control group reflects normal attrition or mortality and the homing failure attributable directly to experimental stress. For instance:

(4) [Control homing failure]=[Natural mortality]+[Homing failure due to experimental stress].

In contrast, homing failure observed in the treatment group is due not only to natural mortality and the homing failure attributable directly to experimental stress, but also to the homing failure that is induced by exposure to pesticides:

(5) [Treatment homing failure]=[Natural mortality]+[Homing failure due to experimental stress]+[Homing failure due to pesticide].

If *m*_hf_ is the homing failure that is solely induced by pesticide treatment, and given Equations 4 and 5, then:

$$\begin{array}{l} m_{\text{hf}}=[\text{Post–exposure homing failure}]\\ \;=[\text{Homing failure due to pesticide}]\\ \;=[\text{Treatment homing failure}]\\ \;-[\text{Control homing failure}],\\ \text{ which is equivalent to Equation 2}. \end{array}$$

In this form *m*_hf_ does indeed **“*[…] estimates the proportion of exposed foragers that might disappear due solely to post-exposure homing failure, all other sources of mortality or homing failure set apart (natural mortality, predation, manipulation stress)*”** as suggested by Henry et al. (supplemental material, 2012; emphasis added).

Importantly, since [Control homing success], [Control homing failure], [Treatment homing success], and [Treatment homing failure] are expressed in individuals.day^−1^, *m*_hf_ is also expressed in individuals.day^−1^. In using Equation 2 to calculate *m*_hf_, we assume that pesticide exposure increases homing failure by a set amount and is *not* proportional to any “normal” homing failure. If so, the *m* parameter of the Khoury et al. (2011) model should be estimated using:

$$m_{\text{pesticide}}=m_{\text{non-exposure}}+m_{\text{hf}}\text{ individuals}\cdot {\text{day}}^{-\text{1}}.$$

## Assumptions and consequences

The choice of Equations 2 or 3 as the basis for the calculation of *m*_hf_ is solely dependent on the assumptions taken. If we choose to use Equation 2, we assume that the homing failure attributable to the exposure of a given dose of pesticide is a fixed value regardless of the “normal” homing failure [assumption (a)]. In this case we assume that most of the homing failure observed in the control is due to natural predation. However, if we choose Equation 3 we assume that the homing failure attributable to pesticide exposure is not only a fixed value in function of the dose of pesticide, but is proportional to the level of the “normal” homing failure [assumption (b)]. For example, we would assume that most of the natural homing failure is due to an aging population of foragers which are more susceptible to the effects of pesticides in comparison to young foragers. We favor assumption (a), although others may disagree.

**Figure 1:**
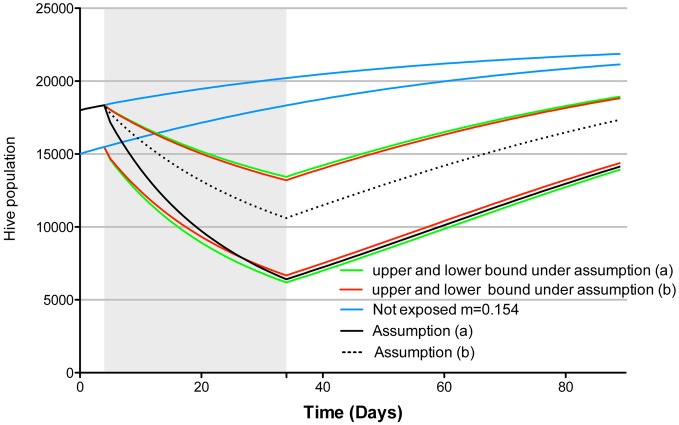
**Simulation of the Khoury et al. (2011) population dynamic model as implemented by Cresswell and Thompson (2012)^2^, model parameters were *w* = 27.000, *L* = 2000, α = 0.25, σ = 0.75 [as per Henry et al. (2012)], starting hive population was 18,000 (lower bound) or 15,000 (upper bound), pesticide exposure condition assumed the extreme case of 100% foragers exposed.** The shaded area corresponds to the exposure period. In blue the projected population growth under a non exposure scenario, in green and red the projected population dynamic under assumptions (a) and (b), respectively, based on Henry et al. (2012) experimental results. Black continuous and dotted line show model projection under assumption (a) and (b) respectively, assuming a normal homing failure of 0.154 individuals.day^−1^, based on experimental results showing 0.7 and 0.42 individuals.day^−1^ homing success in the control and treatment respectively (starting population of 18000 individuals).

Nonetheless, and notwithstanding Guez's (2013) previous critic of Henry et al. (2012), if we use the correct formula as set forth herein, choosing assumptions (a) or (b) has only a minimal impact on Khoury et al.'s (2011) model projection, as exemplified by the green and red curves in Figure 1. However, the difference in model projections that are obtained under assumptions (a) and (b) can become quite significant if the assumed “normal homing failure” (here 0.154 individuals.day^−1^) is far removed from the experimentally determined control homing probability. For example, let us imagine that due to a heightened experimental stress the control homing probability is only 0.7 individuals.day^−1^ and the treatment homing probability is 40% less (i.e., 0.42 individuals.day^−1^). If we use assumption (a), after 30 days of exposure we project more than 4000 less individuals in the hive than if we use assumption (b) (see Figure 1, continuous and dotted black lines). Thus, our assumptions are not without consequence on our model projections, highlighting the need for researchers to explicit all assumptions, and to describe exactly how such assumptions would impact upon outputs within the model used. This is particularly important for models projections that claim to have direct ecological significance, such as the one presented by Henry et al. (2012). Without this crucial information and an understanding of the consequences of various assumptions on model projection, model end points cannot be accurately interpreted and thus cannot be used for accurate or meaningful decision making in the field.

## Conclusion

This contribution highlights the influence of assumptions on model projections and the importance of making explicit not only any assumptions, but also how these influence outputs within a given model. It also highlights that the formula used to calculate *m*_hf_ in Henry et al. (2012) (Equation 1), is conceptually flawed since it calculates the proportional decrease in post exposure homing success given the control instead of the proportional increase in post exposure homing failure. These two values are not numerically equivalent and thus, even without taking into account Guez's (2013) critique, the model projections presented by Henry et al. (2012) are erroneous and cannot be used as a realistic evaluation of the potential impact of dietary thiamethoxam exposure on foraging honeybees. Furthermore, if as Henry et al. (2012) claimed *m*_hf_ was intended to solely represent the post-exposure homing failure: “*all other sources of mortality or homing failure set apart (natural mortality, predation, manipulation stress)*,” *m*_hf_ should have been calculated using Equation 3 as previously described in Guez (2013). Nonetheless, and regardless of the assumptions made by Henry et al. (2012) when calculating *m*_hf_, the *m*_hf_ value presented by Henry et al. (2012) is erroneous as the equation used to calculate it is conceptually flawed. Thus, Henry et al.'s (2012) model projection should not be used as the basis for any meaningful regulatory decision making about the potential risks posed by dietary thiamethoxan intoxication in honeybees.

## Acknowledgment

I would like to thank C. Conway for her feedback during the elaboration of this paper.

## Footnotes

^2^Cresswell and Thomson implementation is available at is available online from the Exeter Research and Institutional Content archive (ERIC) at http://hdl.handle.net/10036/3648. To run simulation under assumption (a) [*m*_pesticide_ = *m*_non − exposure_ + (*m*_non − exposure_ × *m*_hf_)] one need to modify how the parameter *m* is calculated. To this end the formula contained in cell B3 should be changed to “= background + (B16 × background).”
